# Study of short lactation in Sahiwal cattle at organized farm

**DOI:** 10.14202/vetworld.2015.690-694

**Published:** 2015-05-31

**Authors:** U. S. Narwaria, R. K. Mehla, K. K. Verma, S. S. Lathwal, Rajnarayan Yadav, A. K. Verma

**Affiliations:** Livestock Production and Management Section, National Dairy Research Institute, Karnal, Haryana - 132 001, India

**Keywords:** correlation, non-genetic factors, Sahiwal cattle, short lactation

## Abstract

**Aim:**

The aim was to study the associated factors and extent of short lactations in Sahiwal cattle maintained under organized herd.

**Materials and Methods:**

The present study was conducted on Sahiwal cattle (n=530), utilizing 1724 lactation records with respect to lactation length (LL), spread over a period of 15 years (1997-2011), maintained at Livestock Research Center, National Dairy Research Institute, Karnal. Observations of LL were analyzed by descriptive statistical analysis in order to know the extent of short lactation of animals in the herd. Paternal Half sib method was used to estimate the genetic parameters, i.e., heritability, genetic, and phenotypic correlation. The influence of various non-genetic factors (season of calving, the period of calving, parity, type of calving, and season of drying) on LL was studied by least squares analysis of variance technique.

**Results:**

The least squares means for LL was found to be 215.83±3.08 days. Only 32.48% of total lactation records were fell in the range of 251-350 days of LL, while more than three-fourth (76%) of total observations were failed to reach the standard level of 305 milking days. LL class ranges from 251 to 300 days accommodated maximum number of observations (19.2%). The heritability estimate of LL was 0.22±0.07. Positive correlations were found between LL and service period, LL and 305 or less days milk yield, LL and calving interval; whereas dry period was negatively correlated with the LL. The least squares analysis had shown that LL was significantly (p<0.01) influenced by the period of calving, type of calving, and season of drying. Significantly higher LL (276.50±7.21 days) was found in animals calved in the first period than those calved in other periods. The cows dried during summer season had the shortest LL (188.48±7.68 days) as compared to other seasons.

**Conclusion:**

Present findings regarding short lactations occurrence may be alarming for the indigenous herd, demanding comprehensive study with the larger data set. Since LL was influenced by various environmental factors suggesting better managerial tools, besides special attention on the milch animals going to dry during the summer season.

## Introduction

In the present scenario, dairying has emerged as a constant source of income for millions of rural families round the year thus plays a significant role in the Indian agricultural economy. Sahiwal is the best milch breed in the tropics including India [1]. This dairy breed is well known for its higher milk production, greater endurance for hot climate under tropics and subtropics, resistance to the tropical diseases, low cost of maintenance with high feed conversion efficiency and for production of synthetics around the globe [2].

Though India is top milk producer (133 million tons) in the world [3] but the average daily milk production of our indigenous cattle is very poor (2.14 kg) as compared to cross bred and exotic cows (6.87 kg) [4]. This might be attributed to low genetic potential coupled with the various environmental factors primarily poor level of nutrition and improper management.

It has been stated that most of the indigenous cattle were found with average lactation length (LL) below 305 days [5]. However, a study over 40 year lactation data in Sahiwal cattle (n=5897) has shown that LL is decreased by 2 days per year [6] and Ethiopian dairy cows showed negative annual genetic gains of about - 3.348 days for lactation period [7]. Lower milk production may be because of shorter milking period of our indigenous cows.

Therefore, it is most important to improve the production performance of our native stock to meet the expanding demand of milk and milk products for vigorously growing the population of our country. Keeping the above intricacies, the present study has been designed to assess the probable causes and extent of short lactations in Sahiwal cattle maintained under organized herd.

## Materials and Methods

### Ethical approval

The present investigation did not require ethical approval as only phenotypic records obtained from history sheets were used for investigation.

The present investigation was conducted on Sahiwal cattle (n=530) maintained at Livestock Research Centre of National Dairy Research Institute, Karnal, Haryana. Study area is located at 29°42′N latitude and 72° 02′E longitude with an altitude of 250 m above the mean sea level in the bed of Indo-Gangetic alluvial plain. There are four major seasons in the year *viz*. winter (December to March), summer (April to June), rainy (July to September), and autumn (October and November). A subtropical climate with maximum air temperature during summer about 45-48°C and minimum temperature during winter near to 1-4°C prevails in the area. In the study area relative humidity ranges between 41% and 85% and annual rainfall between 760 mm and 960 mm.

### Source of data

The data for the present study were collected from history sheets of Sahiwal cattle maintained at dairy cattle breeding Division of National Dairy Research Institute, Karnal. The data comprising of 1724 lactation records of Sahiwal cattle (n=530) spread over a period of 15 years (1997-2011) were utilized for this study. Incomplete lactations, i.e., transfer, sale or death of an animal during lactation were not included in the present study.

Observations of LL were analyzed by descriptive statistical analysis in order to know the extent of short lactation of animals in the herd. For this purpose, all lactation records were grouped into different classes, keeping a class interval of 50 days. Paternal half-sib method, as described by Becker [8], was used to estimate the genetic parameters, i.e., heritability, genetic, and phenotypic correlation. The sires with three or more number of progeny were included for the estimation of heritability. The data adjusted for significant effects of non-genetic factors were used for estimation of heritability. The influence of various non-genetic factors (season of calving, period of calving, parity, type of calving, and season of drying) on LL was studied by least squares analysis of variance, using the technique described by Harvey [9]. Duncan’s multiple range tests, as modified by Kramer [10], was used for testing differences among least squares means. Each year was divided into four seasons: Winter (December to March), summer (April to June), rainy (July to September), and autumn (October to November). In order to examine the effect of periods of calving, all lactation records were classified into 5 periods of 3 consecutive years (Table-1). On the basis of type of calving, all data were classified into two group namely normal calving and abnormal calving. Abnormal calving included the cases of dystocia and retention of the fetal membrane. Owing to less number of observations after 5^th^ parity, further lactation records were grouped together in a single class.

**Table-1:** Least squares means±SE of LL in Sahiwal cattle.

Sources of variation	LL (in days)	

	N	Mean±SE
Season of calving		
Winter	773	210.25±4.00
Summer	462	222.54±4.93
Rainy	317	225.06±5.78
Autumn	172	205.86±8.00
Period of calving		
1997-1999	183	276.50±7.21^a^
2000-2002	307	206.73±6.16^b^
2003-2005	373	182.60±5.93^c^
2006-2008	403	222.11±5.66^b^
2009-2011	458	219.23±4.98^b^
Parity		
1^st^	487	205.98±5.32
2^nd^	405	212.75±5.45
3^rd^	305	221.53±5.92
4^th^	204	216.50±7.35
5^th^	139	232.09±8.45
6^th^ and above	184	224.35±7.47
Type of calving		
Normal	1634	217.52±3.17^a^
Abnormal	90	185.21±11.48^b^
Season of drying		
Winter	570	222.27±7.07^b^
Summer	422	188.48±7.68^c^
Rainy	404	214.25±7.47^b^
Autumn	328	241.78±7.88^a^
Overall	1724	215.83±3.08

Means bearing same superscript did not differ significantly, LL=Lactation length, SE=Standard error

### Statistical models

For heritability:

Y_ij_ = μ + S_i_ + e_ij_

where,

Y_ij_ = Adjusted value of j^th^ progeny of i^th^ sire

μ = Overall mean

S_i_ = Effect of i^th^ sire

e_ij_ = Random error, NID (0, σ_e_^2^)

For least squares analysis:


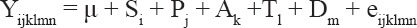


where,


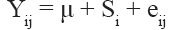


μ = Overall mean

S_i_ = Effect of i^th^ season of calving

P_j_ = Effect of j^th^ period of caving

A_k_ = Effect of k^th^ parity

T_l_ = Effect of l^th^ type of calving

D_m_ = Effect of m^th^ season of drying

e_ijklmn_ = Random error, NID (0, σ_e_^2^)

S_i_, P_j_, A_k_, T_l_, and D_m_ are fixed effects

## Results and Discussion

Least squares analysis revealed an average LL of 215.83±3.08 days, which is far below than the standard 305-days of milking days. Lower LL values were recorded as 204.33±70.35 days reported in local cows in Ethiopia [11]; 213.90±13.74 days in Deoni cows in Marathwada region [12]; 226.98 days in Red Sindhi dairy cattle in Pakistan [13]; 235±1.4 days in Sahiwal cows in Pakistan [14]; 240±5.5 days in crossbred dairy cows in Pakistan [15]; 248±67 days in Sahiwal cattle in Pakistan [5]; and 259.6±6.2 days in Red Chittagong Cattle in Bangladesh [16]. The mean LL of Sahiwal cattle in this study was smaller than that of reported by other authors [17-19].

### Descriptive statistics findings

It was quite clear from Table-2, around 44% of the total observations fell within 200 days of lactation period and more than three-fourth observations (76%) failed to reach the standard level of 305 milking days indicating existence of short lactation in the indigenous cattle herd. Bajwa *et al*. [5] stated that most of the indigenous cattle were found with average LL below 305 days. Higher estimates of LL (290.41±6.29 days) in the same herd of Sahiwal cattle were also reported by Manoj *et al*. [20] that may be due to exclusion of lactation records having LL <100 days. However, maximum numbers of observations (19.2%) among the LL classes were falling within the LL class range of 250-300 days; whereas, around 53% of total observations had LL more than 250 days supporting the inherent capability of Sahiwal cows to exhibit longer milking period.

**Table-2:** Descriptive statistics of LL having class interval of 50 days.

Class range (LL in days)	Number of observations	% of animals	Cumulative %
≤50	152	8.82	8.82
51-100	227	13.17	21.99
101-150	167	9.69	31.67
151-200	203	11.77	43.45
201-250	237	13.75	57.20
251-300	331	19.20	76.40
301-350	229	13.28	89.68
351-400	95	5.51	95.19
401-450	44	2.55	97.74
>450	39	2.26	100.00

LL=Lactation length

### Genetic parameters

#### Heritability estimate

The heritability estimate of LL was 0.22±0.07. The findings of this study were near to the findings of Goshu *et al*. [18] and Endris *et al*. [21], whose heritability estimates were 0.28±0.12 and 0.26±0.09, respectively. Relatively higher estimates (0.49) of heritability were also reported by Nawaz *et al*. [17] in HF in Pakistan. Whereas Rehman *et al*. [6], Kathiravan [22], and Rehman *et al*. [23] observed lower values of heritability, which were 0.09±0.02, 0.07±0.07, and 0.06±0.01, respectively, in Sahiwal cattle. Lower value of heritability indicated that the major part of the variation in LL was governed by environmental factors; therefore, efficient management of animals during adverse conditions is a key to enhance the milking period of Sahiwal cattle.

### Genetic and phenotypic correlation

Genetic and phenotypic correlation between LL and other traits have been presented in Table-3.

**Table-3:** Genetic and phenotypic correlation between LL and other traits in Sahiwal cattle.

Trait	Genetic correlation	Phenotypic correlation
LL-SP	>1	0.34±0.03**
LL-305DMY	0.85±0.05**	0.74±0.01**
LL-DP	−0.71±0.61	−0.40±0.03**
LL-CI	0.73±0.16**	0.38±0.02**

** Significant at 1% level (p<0.01), LL=Lactation length, SP=Service period, 305DMY=305 or less days milk yield, DP=Dry period, CI=Calving interval

### LL and service period (SP)

In this study, the genetic correlation of LL with SP was non-estimable as the estimate was greater than unity. High and positive estimate of genetic correlation (0.62±0.18) between LL and SP in Sahiwal cattle was observed by Kumar [24]; whereas low and negative value (−0.06±0.47) of genetic correlation was reported by Raja [25]. In this study moderate, positive (0.34±0.03) and significant (p<0.01) phenotypic correlation between these two traits was found. Higher estimates of phenotypic correlation (0.54±0.04) between LL and SP were also observed by Manoj *et al*. [20]; whereas lower values (0.16±0.05) were estimated by Goshu *et al*. [18]. Positive correlation of LL with SP indicates that the decrease in SP would facilitate shorter LL. In case of shorter SP, animal conceived earlier and after few months fetal demand for nutrients increased, which would induce the animal to dry earlier.

### LL and 305 or less days milk yield (305DMY)

Genetic correlation of LL with 305 DMY was obtained high, positive (0.85±0.05), and significant (p<0.01) in the present study. High and positive (0.95±0.09) genetic correlation between above traits were also repsorted by Manoj [20]. Whereas Goshu *et al*. [18] reported low and negative (−0.22±0.40) genetic correlation between the LL and 305 DMY. A high, positive (0.74±0.01), and significant (p<0.01) phenotypic correlation between LL and 305 DMY were observed, which is in accordance with the findings of Manoj [20] and Rehman and Khan [23], whose estimates were 0.67±0.03 and 0.71, respectively. Lower estimates of above correlation were also reported by Goshu *et al*. [18] and Kathiravan [22], whose estimates were 0.23±0.05 and 0.32, respectively. Positive and high correlation between LL and 305 DMY suggested that the selection of animals for one trait may lead to improvement in other trait simultaneously.

### LL and dry period (DP)

The results of the present study revealed that there is high, negative (−0.71±0.61), genetic correlation between LL with DP which is statistically non-significant. Negative genetic correlations between above traits have also reported by Goshu *et al*. [18], Kathiravan [22], and Kumar [24], whose estimates were −0.15±0.34, −0.28±0.94 and −0.23±0.39, respectively; whereas positive estimates of above correlations were obtained by Raja [25] and Moges *et al*. [26], which were 0.63±0.83 and 0.98±0.05, respectively. A negative (−0.40±0.03) and significant (p<0.01) phenotypic correlation between LL and DP were found. Negative phenotypic correlation between above traits were also reported by Goshu *et al*. [18] and Kumar [24], whose estimates were −0.42±0.04 and −0.23±0.25, respectively. Contrary to this, Kathiravan [22] and Moges *et al*. [26] obtained positive phenotypic correlation, which were 0.164 and 0.11±0.03, respectively, between these traits. In this study, negative correlations between LL and DP were due to both genetic and environmental reasons; and any efforts in the direction of reducing DP through improvement in management or environmental conditions may improve the LL in the herd.

### LL and calving interval (CI)

Genetic correlation of LL with CI was obtained high, positive (0.73±0.16), and significant (p<0.01). High and positive estimates of above correlation were also reported by Moges *et al*. [26] and Kumar [24], whose estimates were 0.99±0.02 and 0.68±0.22, respectively. A Positive (0.38±0.02) and significant (p<0.01) phenotypic correlation between these traits were found, which is supported by Manoj *et al*. [20] and Kumar [24], whose estimates were 0.53±0.04 and 0.49±0.24, respectively. These positive and higher values of correlations indicated that longer LL was associated with longer CI due to both genetic and environmental reasons, and any increase in LL may result in increased CI.

### Non-genetic factors

#### Season of calving

The results of the present study revealed that the influence of season of calving on LL was statistically non-significant. Similar findings were also reported by Habib *et al*. [16], Nawaz *et al*. [17] and Raja [25]; whereas significant effect of season of calving on LL was observed by other authors [5,10,12].

#### Period of calving

It was observed that the effect of a period of calving on LL was statistically significant (p<0.01), which is supported by most researchers [14,15,19]. However, contrary to the findings in this study, the non-significant effect of period of calving on LL was reported by Habib *et al*. [16] and Endris *et al*. [21]. The average LL was highest (276.50±7.21 days) for the 1^st^ period (1997-1999) and lowest (182.60±5.93 days) for the period of 2003-2005 (Table-1). Average LL of the first period (1997-1999) was highest and significantly different from other periods; the variation in LL observed in different periods reflected the level of management as well as environmental effects. The level of management varies according to the ability of the farm manager, his efficiency in the supervision of the labor, system of crop husbandry, method and intensity of culling and use of financial resources.

#### Parity

The effect of parity on LL was non-significant, which is supported by Habib *et al*. [16]; whereas the significant influence of lactation number on LL was reported by Rehman and Khan [14].

#### Type of calving

The results of the present study revealed that the effect of type of calving on LL was statistically significant (p<0.01). Significantly higher LL (217.52±3.17 days) was observed in normal calver than cows with abnormal calving (185.21±11.48 days) which might be due to uterine disorders and low body condition owing to poor feed intake in affected cows (Table-1).

#### Season of drying

Effect of season of drying was found to be significant (p<0.01) on LL. Animal which dry during the autumn season were reported with significantly longer LL (241.78±7.88 days) as compared to those which dry in summer and rainy seasons (188.48±7.68 and 214.25±7.47 days, respectively) (Table-1). The findings of this study indicates that summer and rainy seasons induced animals to dry earlier that might be due to hot and humid climate coupled with poor hygienic conditions and poor availability of quality feed and fodder. Therefore, early drying of milch animals during hot-dry and hot-humid seasons could be prevented by means of sanitary measures and proper feeding of animals during cool hours of the day for better dry matter intake and to reduce heat load in affected animals.

## Conclusion

The findings of this study reflected that short lactation problem may be alarming for the indigenous herd. Since LL is mainly influenced by environmental factors, therefore proper management measures and good feeding practices are of paramount importance to reduce the occurrence of short lactation in Sahiwal cows. Furthermore, a study on large-scale data is pertinent to assess the problem of short lactation in real sense, which may pave the way for ameliorative actions needed to increase milking days of indigenous milch animals in years to come.

## Authors’ Contributions

USN, RKM, and SSL designed the study. USN conducted the study and analyzed the data. RNY contributed in the data collection. KKV and AKV revised the manuscript. All authors read and approved the final manuscript.
